# Assessing disintegration effectiveness: A thorough evaluation using the SeDeM-ODT expert system for doxylamine succinate orodispersible formulation

**DOI:** 10.1371/journal.pone.0310334

**Published:** 2024-09-17

**Authors:** Salman Ashfaq Ahmad, Syed Muhammad Farid Hasan, Duaa Bafail, Saira Faraz Shah, Muhammad Imran, Tuba Sahar, Azfar Athar Ishaqui

**Affiliations:** 1 Faculty of Pharmacy and Pharmaceutical Sciences, Department of Pharmaceutics, University of Karachi, Karachi, Pakistan; 2 Faculty of Pharmacy, Iqra University, Karachi, Pakistan; 3 Faculty of Medicine, Department of Clinical Pharmacology, King Abdulaziz University, Jeddah, Saudi Arabia; 4 Department of Pharmacy, Nazeer Hussain University, Karachi, Pakistan; 5 Department of Clinical Pharmacy, College of Pharmacy, King Khalid University, Abha, Saudi Arabia; Dow University of Health Sciences, PAKISTAN

## Abstract

**Background:**

The SeDeM-ODT expert system is designed to assess the suitability of the pharmaceutical ingredients for their conversion into an orodispersible formulation by direct compression. The tool can be utilized to select the most appropriate excipients that improve the compressibility and buccodispersibility of the formulation.

**Objective:**

This study aimed to utilize the SeDeM-ODT expert system to evaluate the performance of superdisintegrants and select an appropriate superdisntegrant for Doxylamine Succinate orodispersible formulation.

**Method:**

The SeDeM-ODT expert system scrutinized the excipients to develop an orodispersible Doxylamine Succinate formulation. Among the 15 parameters of the tool, some of them were determined through experimental work, while the remaining were calculated through the experimental values of other parameters. The central composite design approach was used for formulation development. The prepared powder blends were compressed using the direct compression method and evaluated for different parameters (hardness, thickness, diameter, friability, weight variation, water absorption ratio, wetting time, and disintegration time).

**Results:**

The results of the SeDeM-ODT expert system were correlated with the values obtained by the post-compression tests. The Crospovidone formulation (F7) was found to be an optimized formulation as it disintegrated quickly compared with the other formulations containing other superdisintegtrants. The results perfectly endorsed the SeDeM-ODT expert system evaluation, as Crospovidone showed the highest IGCB value of 6.396.

**Conclusion:**

The study observed the effectiveness of the expert system in accurately examining the performance of disintegrating agents. The study observed the effectiveness of the expert system in accurately examining the performance of disintegrating agents. The assessment proved Crospovidone to produce quicker disintegration in Doxylamine Succinate orodispersible formulation.

## 1. Introduction

In the past, extensive experimental work was required to optimize the powder blend’s flow, compressibility, and disintegration behavior; this increased the time and cost of formulation development. There is always a need for such pre-formulation tools that can elucidate the behavior of the powder/powder blend, guide the selection of excipients, and speed up the formulation development process. The SeDeM-ODT expert system is one such tool that has assisted formulators and researchers in examining the critical quality attributes of the powder/powder blend, affecting the quality features of the orodispersible tablet (ODT) formulations. As a pre-formulation technique, the expert system is applied to characterize powder substances based on various parameters related to flow, compressibility, and disintegration behavior. The physical profile of the powder substance is developed, suggesting its suitability for direct compression and buccodispersibility [1–4]. Initially, the SeDeM expert system was used to characterize powder substances based on their rheological characteristics and compressibility. The SeDeM expert system could only provide an estimate of the suitability of the powder substance for direct compression [1,3]. With development, the SeDeM-ODT expert system was created, a modified version of the SeDeM expert system. The modified assessment tool could simultaneously evaluate the powder substances for rheological characteristics, compressibility, and disintegration behavior [5].

The older version of the SeDeM had twelve parameters divided into five indices. In contrast, the incorporation of the sixth index of disgregability resulted in the new and refined version of the SeDeM system, known as the SeDeM-ODT expert system. The new tool has three additional parameters that enhance the capacity of the expert system to characterize disintegration behavior [6]. Overall, the parameters included in the SeDeM-ODT expert system are bulk density, tapped density, inter-particle porosity, Carr’s index, cohesion index, Hausner ratio, angle of repose, powder flow, loss on drying, hygroscopicity, particle size lower than 50 μm, homogeneity index, effervescence, disintegration with disc, and disintegration without disc [7,8]. What differentiates the SeDeM-ODT expert system from the older version is the ability of the new tool to link the suitability of the pharmaceutical ingredient for direct compression and orodispersibility (buccodispersibility). The expert system for ODT formulations is based on Quality by Design (QbD) ICH Q8 guidelines [9] and efficiently assesses the critical quality attributes of the final product [6].

The development of an orodispersible tablet (ODT) formulation has ameliorated patient compliance and adherence among the various drug delivery technologies. Furthermore, ODTs offer many advantages, including improved bioavailability compared to immediate release tablets and capsules, increased stability compared to liquid formulations, and rapid onset of action. These attributes render them superior to conventional oral formulations like immediate release tablets, capsules, and oral liquids, particularly in certain patient types and conditions (such as pediatric and geriatric populations, psychologically unfit, and physiologically and neurologically impaired patients) [10,11]. An ODT refers to a kind of medication that is designed to be disintegrated in the mouth. The Food and Drug Administration (FDA) considers ODT a solid preparation administered orally and disintegrating inside the oral cavity. Moreover, based on the United States pharmacopeial testing method for disintegration, the FDA states an in vitro disintegration time of 30 s or less for ODTs [12]. European Pharmacopeia recommends that the orodispersible tablets disintegrate within 3 min before swallowing [13,14]. Once in the mouth, the formulation quickly releases the active pharmaceutical ingredient (API), resulting in a fine suspension or solution in the saliva. Orodispersible tablets enhance patient compliance by being quickly ingested without chewing or drinking water. Additionally, they ensure precise dosing compared to liquid medication forms [15–17].

Doxylamine Succinate is a multifaceted medication recognized for its various pharmacological effects. Chemically, Doxylamine, categorized as Ethanolamine, competitively binds with the H_1_ receptors, resulting in antagonism. These histaminic receptors are situated in multiple locations, including the uterus, gastrointestinal tract, bronchial muscles, large blood vessels, etc. The sedating effect of the drug is linked to its action on the central and peripheral receptors. Doxylamine Succinate is primarily utilized for the short-term treatment of insomnia because of its sedative properties. Moreover, owing to its antiemetic action, it is also included in the formulations prepared for morning sickness, a condition specifically associated with pregnant women [18–21]. Due to signal transmission inhibition to the vomiting center in the medulla oblongata, nausea and vomiting are antagonized [22].

The presence of superdisintegrants is responsible for the rapid disintegration of orodispersible tablets [23]. Superdisintegrants belong to the class of excipients that promote the breakage of a compressed tablet matrix into fine particles once the dosage form is in contact with the aqueous media. These specialized agents perform this process of mechanical disintegration through various mechanisms, including wicking and swelling, which primarily affect tablet disintegration. Additionally, other methods, such as heat of wetting, deformation recovery, particle repulsion theory, and gas evolution, may contribute to the disintegration of particulate tablets [24,25]. The dissolution process of tablets containing sparingly soluble drug substances is typically hindered by the poor wettability of the tablet, resulting in slow liquid penetration into the tablet matrix. Hence, an increased disintegration time causes delayed release of the drug. Incorporating disintegrants resolves this problem [26]. Disintegrating agents could be of natural or synthetic origin [25].

This study focused on utilizing the SeDeM-ODT expert system to evaluate the effectiveness of the superdisintegrants in producing a quicker disintegration effect. The evaluation will ultimately assist in selecting an appropriate superdisintegrant for the orodispersible formulation of Doxylamine Succinate.

## 2. Materials and methods

### 2.1. Materials

Doxylamine Succinate (USP-NF: Doxylamine Succinate) was purchased from Harika Drugs Private Limited (Hyderabad, India). Povidone K30 (BP, PhEur, USP-NF: Povidone) was purchased from Boai NKY Pharmaceuticals Limited (Jiaozuo, China), Crospovidone (BP, PhEur, USP-NF: Crospovidone) was purchased from Jiaozuo Zhongwei Special Products Pharmaceutical Co. Limited (Jiaozuo, China), Guar Gum (USP-NF: Guar Gum) was purchased from Liberty Natural Products (Oregon, OR, USA), Avicel PH 102 (BP, USP-NF: Microcrystalline Cellulose) was purchased from Sigachi Industries Limited (Hyderabad, India), Sodium Saccharin (BP, PhEur, USP-NF: Saccharin) was purchased from Shanghai Shinesino Biotechnology Co. Limited (Shangai, China), Mannitol (BP, PhEur, USP-NF: Mannitol) was purchased from Hunan Jiudian Hongyang Pharmaceutical Co. Limited (Changsha, China), Aerosil (USP-NF: Colloidal Silicon Dioxide) was purchased from Hubei Huifu Nanomaterial Co. Limited (Yichang, China), and the remaining three excipients, i.e., Sodium Starch glycolate (BP, PhEur, USP-NF: Sodium Starch Glycolate), Croscarmellose Sodium (BP, PhEur, USP-NF: Croscarmellose Sodium), and Magnesium Stearate (BP, PhEur, USP-NF: Magnesium Stearate) were purchased from Vasa Pharmachem Private Limited (Ahmedabad, India). All the ingredients were pharmaceutical grade.

### 2.2. Characterization of excipients and active using SeDeM-ODT expert system

The suitability of the powder blend for undergoing direct compression and conversion into an orodispersible formulation was assessed through the SeDeM-ODT expert system. This suitability determination was based on specific indices, including dimension, compressibility, flowability, stability, dosage, and disgregability, which were determined through the tool. Each index consisted of some parameters; overall, 15 parameters were evaluated through experimental work and mathematical equations. The parameter values obtained via the SeDeM-ODT expert system were converted into radii using appropriate factors. Table 1 lists all the details of the SeDeM-ODT expert system, including the indices and parameters, acceptable ranges (values), and the conversion factors applied to obtain the desired radius value for each parameter of the tool [3,27]. Some parameters were determined empirically following the stated methodology, while others were derived from the experimental values of other parameters. Various compendial methods described in the European and United States Pharmacopeia were employed for the experimental work [13,28]. The Supplementary Materials details the testing procedures and the mathematical formulae used for the parameters. All tests were performed in triplicate to reduce the chances of variation [29]. The SeDeM-ODT expert system determined the index of good compressibility and buccodispersibility (IGCB) for the candidates (excipients) selected to prepare the formulation. An IGCB value ≥ 5 signifies that the excipient could undergo direct compression, and the resulting tablet will have favorable buccodispersible qualities [5].

**Table 1:** Details of SeDeM-ODT expert system factors and parameters. Reprinted/adapted with permission from [14,42]. 2024 Encarna Garcia.

Factor/Index	Parameter (Symbol)	Unit	Equation	Limits	Applied Factor
Dimension	Bulk Density (D_a_)	g/ml	D_a_ = P/V_a_	0–1	10 V
	Tapped Density (D_c_)	g/ml	D_c_ = P/V_c_	0–1	10 V
Compressibility	Inter Particle Porosity (Ie)	-	(D_c_-D_a_)/(D_c_xD_a_)	0–1.2	10 V/1.2
	Carr’s Index (IC)	%	100x(D_c_-D_a_)/D_c_	0–50	V/5
	Cohesion Index (Icd)	N	Experimental	0–200	V/20
Flowability/Powder Flow	Hausner Ratio (IH)	-	D_c_/D_a_	3–1	(30–10 V)/2
	Angle of Repose (α)	°	tan^-1^(h/r)	0–50	10-(V/5)
	Powder Flow (t″)	s	Experimental	0–20	10-(V/2)
Lubricity/Stability	Loss on Drying (%HR)	%	Experimental	0–10	10-V
	Hygroscopicity (%H)	%	Experimental	0–20	10-(V/2)
Lubricity/Dosage	Particles < 50 (%Pf)	%	Experimental	0–50	10-(V/5)
	Homogeneity Index (Iθ)	-	Fm/100 + ΔFmn	0.2x10^-2^	500 V
Disgregability	Effervescence (DE)	min	Experimental	0–5	2(5-V)
	Disintegration with Disc (DCD)	min	Experimental	0–3	3.333(3-V)
	Disintegration without Disc (DSD)	min	Experimental	0–3	3.333(3-V)

### 2.3. Characterization of active (Drug) using SeDeM expert system

The active (drug) ingredient, i.e., Doxylamine Succinate, was evaluated with the SeDeM expert system for its appropriateness for direct compression. The tool was similar to the SeDeM-ODT expert system except for the disgregability index, i.e., the parameters of effervescence, disintegration with the disc, and disintegration without the disc were not part of the SeDeM expert system. Determination of the remaining 12 parameters, conversion of the parameter values into their respective radius, and finally, calculation of the IGC value gave an estimate of the active principle (drug) for its suitability to be directly compressed. An IGC value ≥ 5 suggests that Doxylamine Succinate has the potential to be compressed by the direct compression method. The active drug ingredient was also confirmed for its orodispersible characteristic by subjecting it to three additional parameters of the SeDeM-ODT expert system. The standard for acceptance was similar to the criteria described above for the excipients.

### 2.4. Construction of radar diagrams for the active (Drug) and the excipients

The radii values obtained for each excipient and the active (drug) ingredient (Doxylamine Succinate) were used to construct the radar graphs. The radar diagrams were constructed using Microsoft Excel (version 365). The polygonal area covered in each radar diagram of the excipient indicated its suitability for direct compression and the buccodispersible characteristics of the respective ingredient. In contrast, the radar diagram of Doxylamine Succinate only highlighted its capability to be directly compressed, as the SeDeM expert system was used to evaluate the drug. The radar graphs for the excipients were constructed using 15 parameters of the SeDeM-ODT expert system, whereas 12 parameters assessed for the active moiety (drug) through the SeDeM expert system were utilized to build the radar diagram for Doxylamine Succinate.

### 2.5. Formulae for calculating Parameter Index (IP), Parameter Profile Index (IPP), Index of Good Compressibility (IGC), Index of Good Compressibility and Buccodispersibility (IGCB)

There are different indices included in the SeDeM-ODT expert system. The use of SeDeM-ODT involves determining various parameters, highlighted in Table 1, and then utilizing these parameters to evaluate different indices, which finally helps identify the compressibility and buccodispersibility characteristics of excipients. These indices include parameter index, parameter profile index, index of good compressibility, and index of good compressibility and buccodispersibility. The complete details of these indices are mentioned in Table 2.

**Table 2:** Details of indices used for determining compressibility and buccodispersibility using the SeDeM-ODT expert system.

Index Name	Formula	Description	Acceptable Limit	Reference
Parameter Index (IP)	IP = N° *p* ≥ 5/N° Pt	N° *p* ≥ 5 = number of parameters greater than or equal to 5 and N° Pt = total number of parameters	IP ≥ 0.5	[42]
Parameter Profile Index (IPP)	IPP = average of radii values of all parameters	-	IPP ≥ 5	[42]
Index of Good Compressibility (IGC)	IGC = IPP × Reliability Factor	-	IGC ≥ 5	[42]
Index of Good Compressibility and Buccodispersibility (IGCB)	IGCB = IPP × Reliability Factor	-	IGCB ≥ 5	[14]
Reliability Factor (f)	f = 0.952 (for 12 parameters)	-	-	[42]
	f = 0.971 (for 15 parameters)	-	-	[14]

### 2.6. Formulation development of doxylamine succinate ODTs

The orodispersible tablet formulation of Doxylamine Succinate was developed using a central composite design (CCD) approach with Design Expert® software (version 13). To enhance the robustness and reliability in the optimization process, rotatable CCD was applied with five center points with α value of 1.41421. Multiple center points in the CCD are particularly significant as they augment the ability to investigate the experimental error and the adequacy of the model to represent the responses within the testing region. The design proposed 52 formulations with varying percentages of independent variables.

The direct compression method was utilized for the compression of the orodispersible formulation. The direct compression process involves mixing the formulation ingredients to form a powder blend and then directly compressing the blend. The compression of the tablets was performed on an eccentric single punch machine (Korsch, Berlin, Germany). The target weight was set to 125 mg on the tablet press. Within the permitted ranges, the formulation included the active drug ingredient (Doxylamine Succinate) and the excipients. Multiple literature sources [24,30–35] confirmed the recommended quantities (Table 3). Superdisintegrants of natural (Guar Gum) and synthetic (Crospovidone, Sodium Starch Glycolate, and Croscarmellose Sodium) origin were utilized to enhance the disintegration of the formulation. The disintegrating agent and the binder (Povidone) acted as factors (independent variables). Friability, hardness, wetting time, water absorption ratio, and in vitro disintegration were the responses (dependent variables) evaluated as a result of the variation of independent variables.

**Table 3:** Allowable ranges for excipients used in Doxylamine Succinate orodispersable formulation.

Ingredient (Role)	Supplier	Concentration Used (Allowed Concentration)	References
Povidone (Binder)	Boai NKY Pharmaceuticals Ltd.	1–5% (0.5–5%)	[30]
Crospovidone (Super Disintegrant)	Jiaozuo Zhongwei Special Products Pharmaceutical Co. Ltd.	2–5% (2–5%)	
Sodium Starch Glycolate (Super Disintegrant)	Vasa Pharmachem Pvt. Ltd.	2–5% (2–8%)	
Croscarmellose Sodium (Super Disintegrant)	Vasa Pharmachem Pvt. Ltd.	2–5% (0.5–5%)	
Guar Gum (Super Disintegrant)	Liberty Natural Products	3–8.5% (1–8.5%)	[30–33]
Avicel PH 102 (Binder/Diluent)	Sigachi Industries Ltd.	10.25–24% (20–90%)	[30]
Sodium Saccharin (Sweetener)	Shanghai Shinesino Biotechnology Co. Ltd.	3% (0.25–4%)	[24,34,35]
Mannitol (Diluent)	Hunan Jiudian Hongyang Pharmaceutical Co. Ltd.	37% (10–90%)	[30]
Magnesium Stearate (Lubricant)	Vasa Pharmachem Pvt. Ltd.	2.5% (0.25–5%)	
Aerosil (Glidant)	Hubei Huifu Nanomaterial Co. Ltd.	0.5% (0.1–1%)	

Doxylamine Succinate was mixed with all the excipients, except Aerosil, in a mortar and pestle. Once combined, Aerosil was added, and the complete blend was mixed in a polybag for 5 min. The mode of adding the glidants (e.g., Aerosil) in a formulation affects the surface of solid powdered particles. This effect is not only limited to processing performance but also greatly affects the quality features of the finished product. By coating the solid particle surfaces, particularly the bonding sites, the interparticulate bonding is affected, which has a crucial impact on the tabletabilty of the pharmaceutical formulation [36]. Hence, Aerosil was added and mixed in last after combining the remaining ingredients. The incorporation of Aerosil lastly minimized the interference of Aerosil with bonding, which is crucial for compressibility and compactibility.

### 2.7. Pre-compression tests

The powder blends were evaluated for pre-compression parameters, including determining bulk density, tapped density, Carr’s index, Hausner’s ratio, and angle of repose. The tests were performed as per the pharmacopoeial specifications.

### 2.8. Evaluation of compressed tablets

The formulated orodispersible Doxylamine Succinate tablets were examined for various characteristics to confirm the performance of the SeDeM-ODT expert system. These assessment parameters included hardness, thickness, diameter, weight variation, friability, water absorption ratio, wetting time, and in vitro disintegration time.

#### 2.8.1. Hardness

The hardness represents the tensile strength of the tablet formulation. The hardness of tablets is linked with the physical deformation of the particles in the blend, the role of the binder in the formulation, and the compressional force applied during the compression of the powder blend. Ten tablets from each formulation were randomly selected. Hardness was determined using the Erweka Hardness tester (Fujiwara Seisukusho Corporation, Wakayama, Japan). The crushing strength of the tablet was determined by placing the tablet diametrically on the lower anvil and applying pressure. The values were recorded in Newtons (N) [17,37].

#### 2.8.2. Thickness and diameter

Ten tablets from each batch (F1–F52) were selected at random. The thickness and diameter were determined using a vernier caliper (Seiko, Shanghai, China) [17].

#### 2.8.3. Friability

Removal of fine particles from the surface of the tablets results in a change in the weight of the tablets, as indicated by the value of the friability. Roche friabilator (Basel, Switzerland) was used to find the friability test on the sample of ten tablets of each formulation. A pre-weighed, dedusted sample of tablets was placed inside the friabilator. After 100 rotations, the tablets were weighed again. The difference in weight represented friability. The acceptable limit for weight loss is less than 1%. Friability was calculated using the following formula [17,37]:

$$\%\;Friability=\frac{Initial\;Weight-Final\;Weight}{Initial\;Weight}\times 100$$

#### 2.8.4. Wetting time and water absorption ratio

The method utilized by Bi et al. [38] was employed to determine the wetting time and the water absorption ratio. A piece of tissue paper was folded twice and placed in a small petri dish containing 6 mL of water. The tablet was placed on the tissue paper, and the time the wetting process took to complete was recorded. The weight of the tablet was determined once the wetting was complete [37].

The water absorption ratio (R) was determined using the following mathematical formula [17]:

$$Water\;Absorption\;Ratio=\frac{W_{a}-W_{b}}{W_{b}}\times 100$$

where W_a_ = weight of the tablet after absorption and W_b_ = weight of the tablet before absorption.

#### 2.8.5. In vitro disintegration

An Erweka disintegration tester (Erweka ZT-2, Langen, Germany) was used to evaluate the disintegration time of the tablet samples. Six tablets from each formulation were selected randomly and assessed against the compendial requirements. The disintegration test was performed by placing one tablet in each tube, and the assembly was suspended into a 1000 mL beaker containing distilled water maintained at 37 ± 2°C [37]. The European Pharmacopeia mentions that ODTs must disintegrate within 3 min [13].

## 3. Results

### 3.1. SeDeM-Based characterization of ingredients

**Fig 1:**
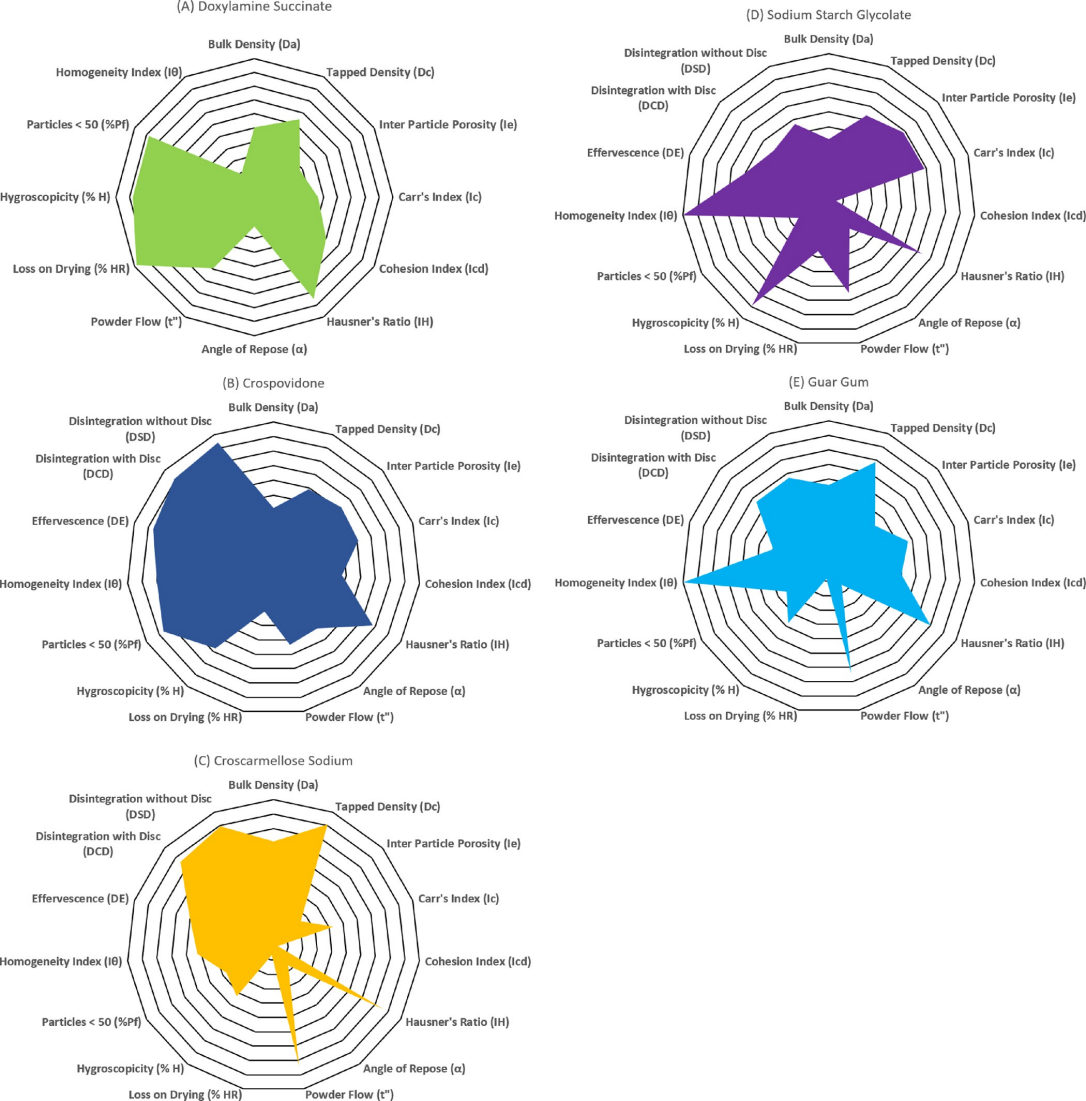
Radar graphs. (A) Doxylamine Succinate: Constructed using 12 parameters of SeDeM expert system; (B) Crospovidone: Constructed using 15 parameters of SeDeM-ODT expert system; (C) Croscarmellose Sodium: Constructed using 15 parameters of SeDeM-ODT expert system; (D) Sodium Starch Glycolate: Constructed using 15 parameters of SeDeM-ODT expert system; and (E) Guar Gum: Constructed using 15 parameters of SeDeM-ODT expert system.

The study used the SeDeM-ODT expert system to assess the compressibility and buccodispersibility of various excipients, including superdisintegrants (natural and synthetic superdisintegrants), Mannitol, Sodium Saccharin, and Microcrystalline Cellulose. The tool mainly focused on assessing superdisintegrants to identify and select the most appropriate superdisintegrant for the orodispersible formulation. The SeDeM-ODT expert system’s evaluation framework incorporated 15 parameters, which are outlined in Table 1. The parameters were determined using a combination of calculative and experimental procedures, as highlighted in Table 1 and Supplementary Materials. The quantitative data collected for each ingredient enabled the determination of the parameter index (IP), parameter profile index (IPP), and the index of good compressibility and buccodispersibility (IGCB), as shown in Table 4. The numerical values obtained from all the parameters were converted into their respective radii using particular mathematical factors detailed in Table 1 to enable a thorough study. The radii were graphically represented using radar charts, as seen in Fig 1. Using the IGCB values and the polygonal area depicted in radar graphs for the excipients, this analytical method helped to characterize the studied ingredients thoroughly.

**Table 4:** SeDeM-ODT expert system parameters.

Content	Da	Dc	Ie	Ic	Icd	IH	α	t"	% HR	% H	% Pf	Iθ	DE	DCD	DSD	IP	IPP	IGC	IGCB
Drug	0.501	0.650	0.458	22.923	120.000	1.297	39.220	8.100	0.190	2.480	6.100	0.004	5.000	3.000	3.000	0.667	5.981	5.694	4.646
PO	0.523	0.680	0.441	23.088	130.000	1.300	11.500	6.300	0.780	9.200	6.000	0.004	2.500	1.700	1.100	0.733	6.064	-	5.888
CCS	0.714	0.909	0.300	21.452	5.700	1.273	41.700	3.250	9.360	11.450	31.450	0.011	2.000	0.433	0.317	0.533	5.290	-	5.137
SSG	0.416	0.633	0.824	34.281	9.600	1.522	38.000	7.000	6.450	2.150	38.000	0.020	2.000	1.483	1.300	0.667	5.506	-	5.346
MCC	0.486	0.642	0.500	24.299	138.000	1.321	39.380	11.000	7.400	0.950	33.420	0.016	4.000	2.830	3.000	0.333	4.549	-	4.417
CP	0.410	0.590	0.744	30.508	94.000	1.439	24.560	9.360	7.000	6.480	6.660	0.016	0.666	0.267	0.183	0.800	6.587	-	6.396
GG	0.562	0.787	0.508	28.563	100.000	1.400	42.500	5.200	9.180	10.560	33.400	0.020	3.000	1.000	1.000	0.600	5.435	-	5.277
MN	0.668	0.810	0.263	17.541	180.000	1.213	24.000	12.000	2.600	0.900	19.000	0.014	4.000	1.000	1.000	0.733	6.206	-	6.026
SS	1.000	1.200	0.167	16.667	200.000	1.200	36.000	8.500	0.200	2.500	1.000	0.010	1.200	1.100	1.100	0.800	7.193	-	6.984

Drug = Doxylamine Succinate, PO = Povidone, CCS = Croscarmellose Sodium, SSG = Sodium Starch Glycolate, MCC = Microcrystalline Cellulose, CP = Crospovidone, GG = Guar Gum, MN = Mannitol, SS = Sodium Saccharin, IP = Parameter Index, IPP = Parameter Profile Index, IGCB = Index of Good Compressibility and Buccodispersibility

The results outlined in Table 4 revealed that among the four types of disintegrating agents, Crospovidone has the highest IGCB value (IGCB = 6.396). In contrast, Croscarmellose Sodium, Sodium Starch Glycolate, and Guar Gum had IGCB values of 5.137, 5.346, and 5.327, respectively. Although Doxylamine Succinate was cleared for the compressibility criteria once it gave the value of IGC ≥ 5, its lower disgregability characteristic made the therapeutic agent inappropriate for an orodispersible formulation. Hence, adding a suitable superdisintegrant in an appropriate quantity helped improve the orodispersibility of the therapeutic moiety.

### 3.2. Formulation development and assessment of disintegration effectiveness

Direct compression of CCD proposed formulations produced tablets, each weighing 125 mg and consisting of 20% API (Doxylamine Succinate). The blends were subjected to a pre-compression examination. The examination included determining bulk density, tapped density, Carr’s index, Hausner’s ratio, and angle of repose. As shown in Table 5, all the powder blends (F1–F52) showed excellent flow characteristics, as the average value of the angle of repose ranged from 26.990° to 30.044° [39], whereas formulations F1–F26 exhibited better compressibility owing to lower Carr’s index values in contrast to higher values for formulations F27–F52.

**Table 5:** Pre-formulation test values (average values).

Formulation	Bulk Density (g/ml)	Tapped Density (g/ml)	Carr’s Index (%)	Hausner Ratio	Angle of Repose (°)
Crospovidone Formulations (F1-F13)	0.387 ± 0.001	0.444 ± 0.002	12.895 ± 0.537	1.148 ± 0.007	26.990 ± 0.011
Sodium Starch Glycolate Formulations (F14-F26)	0.427 ± 0.001	0.489 ± 0.001	12.675 ± 0.127	1.145 ± 0.002	28.736 ± 0.024
Croscarmellose Sodium Formulations (F27-F39)	0.382 ± 0.001	0.472 ± 0.001	19.057 ± 0.314	1.235 ± 0.005	31.967 ± 0.033
Guar Gum Formulations (F40-F52)	0.489 ± 0.001	0.615 ± 0.002	20.462 ± 0.297	1.257 ± 0.005	30.044 ± 0.517

The compressed formulations were assessed for various parameters, as shown in Table 6. The determination of post-compression parameters helped identify an optimized formulation that fulfills the requirements of an orodispersible formulation. Moreover, these tests also helped in assessing the performance of the disintegrants, which gave a comparison among the four different superdisintegrants used in the study. Formulation containing Guar Gum (F40–F52) as a disintegrating agent resulted in tablets of the lowest hardness ($\bar{x}$ = 2.69 kg/cm2, S.D. = 0.09). Orodispersible formulations containing Crospovidone (F1–F13) produced tablets with an average hardness of 3.43 kg/cm2 ± 0.131, which was the highest hardness recorded among other formulations consisting of Guar Gum, Croscarmellose Sodium, or Sodium Starch Glycolate as the superdisintegrants.

**Table 6:** Post-formulation tests.

Tests	Hardness (N)	Thickness (mm)	Diameter (mm)	Weight Variation (mg)	Friability (%)	Water Absorption Ratio	Wetting Time (seconds)	Disintegration Time (seconds)
F1	3.46 ± 0.212	3.70 ± 0.019	6.00 ± 0.011	Passes	0.65 ± 0.022	101.37 ± 0.420	10.96 ± 0.220	28.52 ± 0.911
F2	3.45 ± 0.310	3.71 ± 0.018	5.91 ± 0.012	Passes	0.65 ± 0.021	101.72 ± 0.312	10.56 ± 0.251	28.50 ± 0.874
F3	3.45 ± 0.122	3.70 ± 0.019	5.90 ± 0.011	Passes	0.66 ± 0.023	101.40 ± 0.495	10.70 ± 0.310	28.50 ± 0.975
F4	3.20 ± 0.210	3.70 ± 0.018	5.92 ± 0.011	Passes	0.58 ± 0.022	101.30 ± 0.325	11.50 ± 0.315	28.92 ± 0.578
F5	3.43 ± 0.511	3.71 ± 0.017	5.91 ± 0.013	Passes	0.67 ± 0.022	101.32 ± 0.321	11.36 ± 0.328	28.90 ± 0.612
F6	3.62 ± 0.610	3.72 ± 0.016	5.90 ± 0.012	Passes	0.67 ± 0.021	101.45 ± 0.422	10.29 ± 0.425	28.32 ± 0.408
F7	3.50 ± 0.091	3.70 ± 0.012	5.90 ± 0.012	Passes	0.67 ± 0.020	101.50 ± 0.433	9.89 ± 0.150	27.00 ± 0.158
F8	3.59 ± 0.100	3.71 ± 0.014	5.90 ± 0.013	Passes	0.67 ± 0.021	101.48 ± 0.352	10.03 ± 0.214	27.55 ± 0.924
F9	3.46 ± 0.221	3.70 ± 0.012	5.91 ± 0.011	Passes	0.63 ± 0.021	102.00 ± 0.310	10.16 ± 0.321	28.12 ± 0.910
F10	3.44 ± 0.213	3.71 ± 0.015	5.91 ± 0.011	Passes	0.65 ± 0.022	101.43 ± 0.510	10.43 ± 0.412	28.45 ± 0.541
F11	3.55 ± 0.210	3.70 ± 0.014	5.92 ± 0.012	Passes	0.66 ± 0.023	101.33 ± 0.440	11.23 ± 0.325	28.88 ± 0.310
F12	3.20 ± 0.356	3.71 ± 0.018	5.91 ± 0.012	Passes	0.59 ± 0.020	101.35 ± 0.210	11.10 ± 0.159	28.75 ± 0.420
F13	3.25 ± 0.335	3.72 ± 0.019	5.92 ± 0.013	Passes	0.60 ± 0.021	102.10 ± 0.110	10.83 ± 0.611	28.50 ± 0.420
F14	2.91 ± 0.187	3.70 ± 0.021	5.90 ± 0.012	Passes	0.48 ± 0.021	99.45 ± 0.112	12.34 ± 0.524	59.76 ± 0.988
F15	3.00 ± 0.250	3.70 ± 0.019	5.90 ± 0.012	Passes	0.50 ± 0.022	99.20 ± 0.210	12.35 ± 0.570	59.80 ± 0.810
F16	3.10 ± 0.293	3.70 ± 0.019	5.90 ± 0.012	Passes	0.50 ± 0.032	99.00 ± 0.110	12.25 ± 0.257	59.23 ± 1.010
F17	3.14 ± 0.563	3.70 ± 0.020	5.91 ± 0.013	Passes	0.52 ± 0.033	99.50 ± 0.405	12.21 ± 0.210	58.00 ± 0.876
F18	3.13 ± 0.425	3.71 ± 0.018	6.00 ± 0.013	Passes	0.52 ± 0.031	99.40 ± 0.302	12.27 ± 0.108	59.23 ± 0.763
F19	3.01 ± 0.578	3.70 ± 0.019	5.91 ± 0.011	Passes	0.51 ± 0.021	99.35 ± 0.326	12.28 ± 0.207	59.23 ± 0.489
F20	3.11 ± 0.892	3.71 ± 0.019	5.90 ± 0.011	Passes	0.50 ± 0.022	99.30 ± 0.417	12.29 ± 0.311	59.23 ± 1.108
F21	3.12 ± 0.727	3.71 ± 0.012	5.92 ± 0.011	Passes	0.51 ± 0.022	99.15 ± 0.119	12.37 ± 0.310	59.82 ± 1.210
F22	3.20 ± 0.325	3.71 ± 0.013	5.91 ± 0.011	Passes	0.51 ± 0.032	99.10 ± 0.109	12.22 ± 0.200	58.50 ± 0.879
F23	3.15 ± 0.112	3.71 ± 0.018	5.91 ± 0.012	Passes	0.51 ± 0.033	99.60 ± 0.415	12.31 ± 0.241	59.23 ± 0.218
F24	3.20 ± 0.112	3.71 ± 0.012	5.90 ± 0.013	Passes	0.53 ± 0.032	99.55 ± 0.610	12.32 ± 0.514	59.50 ± 0.199
F25	3.05 ± 0.223	3.70 ± 0.013	5.92 ± 0.013	Passes	0.51 ± 0.032	99.25 ± 0.514	12.38 ± 0.312	59.85 ± 0.560
F26	3.10 ± 0.789	3.71 ± 0.012	6.00 ± 0.012	Passes	0.48 ± 0.032	99.05 ± 0.328	12.24 ± 0.321	58.50 ± 1.310
F27	3.10 ± 0.620	3.70 ± 0.013	5.90 ± 0.013	Passes	0.51 ± 0.032	100.56 ± 0.521	11.45 ± 0.510	53.00 ± 1.200
F28	3.20 ± 0.451	3.71 ± 0.014	5.91 ± 0.013	Passes	0.52 ± 0.022	100.10 ± 0.219	11.40 ± 0.601	51.25 ± 1.028
F29	3.36 ± 0.369	3.71 ± 0.017	5.93 ± 0.013	Passes	0.51 ± 0.022	100.43 ± 0.217	11.47 ± 0.427	53.00 ± 0.781
F30	3.29± 0.310	3.70 ± 0.017	5.92 ± 0.012	Passes	0.51 ± 0.022	100.60 ± 0.627	11.57 ± 0.311	54.00 ± 1.200
F31	3.25 ± 0.412	3.70 ± 0.015	5.90 ± 0.012	Passes	0.54 ± 0.032	100.52 ± 0.257	11.43 ± 0.307	52.10 ± 1.301
F32	3.13 ± 0.815	3.70 ± 0.015	5.90 ± 0.013	Passes	0.50 ± 0.032	100.39 ± 0.105	11.48 ± 0.109	53.00 ± 1.000
F33	3.10 ± 0.712	3.71 ± 0.012	5.91 ± 0.013	Passes	0.51 ± 0.022	100.35 ± 0.510	11.50 ± 0.401	53.00 ± 0.280
F34	3.11 ± 0.355	3.70 ± 0.012	5.91 ± 0.013	Passes	0.55 ± 0.021	100.31 ± 0.509	11.52 ± 0.350	53.00 ± 0.700
F35	3.02 ± 0.115	3.71 ± 0.012	5.91 ± 0.011	Passes	0.50 ± 0.021	100.22 ± 0.407	11.55 ± 0.245	53.50 ± 0.896
F36	3.10 ± 0.212	3.71 ± 0.015	5.91 ± 0.011	Passes	0.53 ± 0.012	100.27 ± 0.417	11.53 ± 0.140	53.00 ± 0.743
F37	3.00 ± 0.425	3.71 ± 0.013	5.91 ± 0.012	Passes	0.53 ± 0.023	100.47 ± 0.238	11.58 ± 0.215	54.00 ± 0.777
F38	3.20 ± 0.662	3.71 ± 0.013	5.92 ± 0.013	Passes	0.52 ± 0.024	100.18 ± 0.125	11.60 ± 0.300	55.21 ± 1.025
F39	3.25 ± 0.672	3.72 ± 0.012	5.93 ± 0.013	Passes	0.52 ± 0.022	100.14 ± 0.614	11.42 ± 0.149	51.40 ± 1.045
F40	2.61 ± 0.821	3.72 ± 0.013	5.92 ± 0.014	Passes	0.48 ± 0.022	99.03 ± 0.287	12.89 ± 0.325	59.82 ± 0.632
F41	2.70 ± 0.315	3.72 ± 0.012	5.93 ± 0.013	Passes	0.50 ± 0.022	98.77 ± 0.471	12.99 ± 0.348	61.02 ± 0.457
F42	2.59 ± 0.720	3.71 ± 0.013	5.90 ± 0.013	Passes	0.50 ± 0.032	98.73 ± 0.502	13.01 ± 0.487	61.50 ± 0.693
F43	2.68 ± 0.514	3.70 ± 0.018	5.90 ± 0.012	Passes	0.48 ± 0.032	98.70 ± 0.600	13.02 ± 0.259	61.50 ± 0.907
F44	2.68 ± 0.622	3.71 ± 0.018	5.90 ± 0.011	Passes	0.47 ± 0.021	99.00 ± 0.512	12.9 ± 0.257	60.12 ± 1.310
F45	2.69 ± 0.425	3.71 ± 0.017	5.90 ± 0.012	Passes	0.51 ± 0.021	99.12 ± 0.587	12.91 ± 0.745	60.12 ± 1.020
F46	2.70 ± 0.780	3.71 ± 0.019	5.91 ± 0.012	Passes	0.51 ± 0.033	99.10 ± 0.340	12.86 ± 0.124	59.80 ± 1.270
F47	2.70 ± 0.832	3.70 ± 0.017	5.90 ± 0.012	Passes	0.49 ± 0.022	99.07 ± 0.379	12.87 ± 0.258	59.80 ± 0.710
F48	3.00 ± 0.813	3.70 ± 0.017	5.91 ± 0.011	Passes	0.49 ± 0.021	98.83 ± 0.564	12.97 ± 0.218	61.00 ± 0.490
F49	2.68 ± 0.412	3.72 ± 0.015	5.91 ± 0.012	Passes	0.47 ± 0.022	99.23 ± 0.512	12.93 ± 0.458	60.12 ± 0.689
F50	2.66 ± 0.527	3.70 ± 0.015	5.90 ± 0.012	Passes	0.48 ± 0.032	98.00 ± 0.210	12.94 ± 0.297	60.12 ± 0.783
F51	2.67 ± 0.220	3.71 ± 0.012	5.91 ± 0.011	Passes	0.48 ± 0.011	99.05 ± 0.358	12.98 ± 0.478	61.00 ± 1.201
F52	2.70 ± 0.325	3.70 ± 0.013	6.00 ± 0.011	Passes	0.47 ± 0.022	98.87 ± 0.379	12.95 ± 0.641	60.12 ± 1.000

The F7 formulation, containing Crospovidone as a superdisintegrant, was identified as an optimized formulation, showing the lowest disintegration time of 27 s with the water absorption ratio and wetting time of 101.50 and 9.89 s, respectively.

## 4. Discussion

The SeDeM-ODT expert system is an advancement of the SeDeM expert system for developing orodispersible tablets (ODTs). This research utilized this methodology due to its systematic and quantitative approach in evaluating the suitability of the pharmaceutical ingredients for direct compression. Various literature has identified that the SeDeM-ODT expert system helps reduce the number of extra, repetitive laboratory tests since it provides insights into a powder mixture’s rheological and compactable properties for producing conventional tablets through direct compression. Additionally, it offers information that deepens the understanding of formulation design [3,40,41].

The SeDeM expert system assessed the compressibility characteristics of Doxylamine Succinate. The index of good compressibility (IGC) obtained through the tool proved the compression characteristic and appropriateness of the active pharmaceutical ingredient (API), i.e., the drug, for direct compression. An IGC value greater than 5, as in the case of Doxylamine Succinate, represented the suitability of the material to be directly compressed. The active drug ingredient did not produce satisfactory results for buccodispersibility when subjected to the SeDeM-ODT expert system. Characterizing the API through the SeDeM-ODT expert system was not essential, as the active drug principle is usually considered deficient in the disgregability parameter, which improves by adding disintegrating agents [14].

Superdisintegrants were subjected to SeDeM-ODT expert system tests to evaluate their compressibility and buccodispersibility features. Various micromeritic properties obtained for different experimentally determined and calculated values were converted into radii values. These radii values were transformed into radar diagrams. The radar graphs depicted the polygonal diagrams that illustrated the micromeritic properties of the formulation ingredients. The radar graph would be the circumscribed regular polygon if all the radii values were equal to 10. The polygon is termed a dodecagon in the case of Doxylamine Succinate (a drug), and a pentadecagon, in the case of the excipients. The polygonal area of the radar diagram of the drug, constructed using the SeDeM expert system, represented the suitability of the Doxylamine Succinate to undergo direct compression. In contrast, the polygonal area of the radar charts for superdisintegrants, obtained through applying the SeDeM-ODT expert system, showed their appropriateness for conversion into an ODT by direct compression. A larger polygonal area represents the more outstanding suitability of the formulation ingredient [8,14,42]. The radar plots constructed using data obtained from the SeDeM-ODT expert system proved the suitability of Crospovidone in an ODT formulation, as it had the largest shaded polygonal area among the four superdisintegrants.

The determination of the IGCB value further supported and ranked Crospovidone on top (IGCB = 6.396) among the remaining three superdisintegrants, i.e., Sodium Starch Glycolate (IGCB = 5.346), Croscarmellose Sodium (IGCB = 5.137) and Guar Gum (IGCB = 5.237). These findings were in line with the findings of Rao, Sapate and Sonawane [8], in which Crospovidone had the highest IGCB value (5.75) among Sodium Starch Glycolate and Croscarmellose Sodium used in formulation development. Hence, Crospovidone demonstrated ideal dimensional properties, including optimal compressibility, good flowability, required lubricity, and outstanding disgregability. What differed from the mentioned study was the researcher’s finding that Sodium Starch Glycolate has an IGCB value of less than five, representing its lack of buccodispersibility. In contrast, Glycolys® (a brand of Sodium Starch Glycolate) gave an IGCB value above 5 when evaluated through SeDeM-ODT expert system tests by Aguilar-Díaz, García-Montoya, Suñe-Negre, Pérez-Lozano, Miñarro and Ticó [5]. The findings of Aguilar-Díaz, García-Montoya, Suñe-Negre, Pérez-Lozano, Miñarro and Ticó [5] complied with our study, proving its good buccodispersible characteristic.

The evaluation of flowability and compressibility for the prepared powdered blends (F1–F52) judged the effect of the added disintegrant in the formulation. Appropriate flowability was observed for all the formulations, with the angle of repose values ranging from 26.97° to 32.01°. Formulations containing Crospovidone (F1–F14) had the angle of repose values on the lower side of the range, consistent with the research work of Puttewar, Kshirsagar, Chandewar and Chikhale [37] for Doxylamine and Pyridoxine combined formulation. Furthermore, a change of disintegrant in formulations (F1–F52) showed a shift in compressibility behavior, as exhibited by the compressibility index (Carr’s Index) value. The average value of 12.895% ± 0.537 not only resembled the findings of previous research [37] but also supported the current SeDeM-ODT expert system findings, which marked Crospovidone to have the best compressibility and flowability characteristics among the other superdisintegrants under investigation.

Various post-compression parameters evaluated for the compressed Doxylamine Succinate tablets endorsed the results of the SeDeM-ODT expert system and confirmed the tool’s effectiveness for evaluating the disintegrants. Wetting time and water absorption ratio values were used to compare the formulations, which differed due to varying concentrations of Povidone and the type and concentration of superdisintegrants. The average wetting time and water absorption ratio for formulations F1–F13 were 10.695 s ± 0.50 and 101.519 ± 0.250, respectively. These values made the formulations containing Crospovidone (F1–F13) superior to the others (F14–F52). Among the synthetic superdisintegrants, the wetting time of Crospovidone formulations was the lowest, followed by Croscarmellose Sodium and Sodium Starch Glycolate formulations. The wetting time of the natural disintegrant, Guar Gum (F40–F52), was comparable with the Sodium Starch Glycolate formulations. The results for synthetic superdisintegrants closely resembled the findings of Puttewar, Kshirsagar, Chandewar and Chikhale [37], particularly regarding sequence and wetting time values. Moreover, the formulations of Crospovidone in our study exhibited a higher water absorption ratio, consistent with the research outcomes of Puttewar, Kshirsagar, Chandewar and Chikhale [37]. Lower wetting time and a higher water absorption ratio mark Crospovidone as a suitable superdisintegrant for an orodispersible formulation.

Past investigations have compared the disintegration performance of superdisintegrants in various formulations. Comparing the current results with past scientific studies revealed that Crospovidone exhibits quicker disintegration compared to Croscarmellose Sodium and Sodium Starch Glycolate. Yousaf, et al. [43] concluded that adding Crospovidone to Stevia tablets resulted in a faster disintegration time than Croscarmellose Sodium and Sodium Starch Glycolate. Dubey, et al. [44] found that Ibuprofen mouth dissolving tablets with Crospovidone disintegrated faster and released Ibuprofen more quickly than those with Croscarmellose Sodium. The disintegration time for the orodispersible formulations containing Guar Gum was between 60 and 61 s. This time was higher than the disintegration time reported in the literature [32,33].

The research findings suggested the F7 formulation (containing Crospovidone) as an optimized formulation. The formulation exhibited the shortest disintegration time, the lowest wetting time, and the highest water absorption ratio. The formulation showed appropriate hardness and an acceptable friability. The findings affirmed the appropriateness of the SeDeM-ODT expert system in assessing the ingredients and assured the tool’s strength in selecting the correct ingredients that produce promising results.

## 5. Conclusion

The SeDeM-ODT expert system significantly improves the efficiency and predictability of orodispersible tablet (ODT) formulation processes, making a crucial contribution to pharmaceutical technology by aiding in the creation of more efficient and accessible drug delivery systems. The current research witnessed the expert system tool’s efficiency in identifying each excipient’s performance, particularly the superdisintegrants. The assessment tool assessed the suitability of each disintegrant and identified Crospovidone as producing quicker disintegration of the Doxylamine Succinate orodispersible formulation in comparison with other synthetic (Croscarmellose Sodium and Sodium Starch Glycolate) and natural (Guar Gum) disintegrating agents.

## Supporting information

S1 File SeDeM-ODT expert system. Details (method and formula), of 15 parameters included in the expert system are mentioned in the supplementary material.
(DOCX)
