# Supplementary material for: Assessing disintegration effectiveness: A thorough evaluation using the SeDeM-ODT expert system for doxylamine succinate orodispersible formulation
Source: PLoS One. 2024 Sep 17;19(9):e0310334. doi: 10.1371/journal.pone.0310334 (PMC11407626; doi:10.1371/journal.pone.0310334)
Supplement: S1 File — Details (method and formula), of 15 parameters included in the expert system are mentioned in the supplementary material. (DOCX) [file pone.0310334.s001.docx]

**SUPPORTING INFORMATION**

**SeDeM-ODT EXPERT SYSTEM:** There are 15 parameters included in SeDeM-ODT expert system. The details of each parameter are mentioned below:

1. *BULK DENSITY* (Ph Eur 2.9.34) [1]:

The untapped volume, i.e., the bulk volume (V_b_), of the powder filled inside a graduated cylinder was used to determine the bulk density. The following formula was used:

Bulk Density (D_a_) = Mass of powder/Bulk volume

1. *TAPPED DENSITY* (Ph Eur 2.9.34) [1]:

The tapped volume (V_c_) of the powder was measured via a graduated cylinder and then substituted in the following formula to calculate the tapped density:

Tapped Density (D_c_) = Mass of powder/Tapped volume

1. *INTER-PARTICLE POROSITY* [2]: It indicates the void spaces between the powder particles. It was determined with the help of the bulk and the tapped density values. The following formula was used to perform the calculation:

Inter particle porosity (Ie) = (D_c_ - D_a_)/(D_c_ x D_a_)

1. *CARR’S INDEX* (Ph Eur 2.9.34) [1]: The compressibility characteristic of the powder was evaluated with the value of Carr’s Index. It was calculated with the following formula:

Carr’s Index (Ic) = [(D_c_ - D_a_)/D_c_] x 100

1. *COHESION INDEX*: The powder's cohesion index (Icd) was evaluated by compressing the powder into a compact, and then the hardness of the compact was determined in Newtons (N).
2. *HAUSNER RATIO* (Ph Eur 2.9.34) [1]: The values of bulk and tapped density were used to calculate the Hausner ratio. The value gave an idea of the flowability characteristic of the powder. The calculation was done with the following equation:

Hausner Ratio (IH) = D_c_/D_a_

1. *ANGLE OF REPOSE* (Ph Eur 2.9.36) [1, 3]: The angle of repose is defined as the angle of the powder heap with the horizontal surface. A sample of 100 g was passed through a funnel (attached at a fixed height). The height and radius of the powder heap were measured, which helped to determine the angle of repose. The following formula was used to calculate the angle of repose:

tan α = height/radius

1. *POWDER FLOW* (Ph Eur 2.9.16) [1]: The time required by a fixed mass (100 g) of a powder to flow out of the funnel was represented by the powder flow (t”).
2. *LOSS ON DRYING* (Ph Eur 2.2.32) [1]: The loss on drying (% HR) was calculated when a pre-weighed powder sample (10 g) was kept in a convection oven at 105 ± 2°C. The sample was kept in an oven until the change in mass became constant.
3. *HYGROSCOPICITY* [2]: A pre-weighed powder sample (10 g) was kept in a humidifier set at a relative humidity of 76 ± 2% and a temperature of 22°C ± 2% for 24 hours. The hygroscopicity (% H) indicates an increase in the mass of the sample.
4. *PARTICLE SIZE LOWER THAN 50 MICRONS* (Ph Eur 2.9.12) [1]: A 100 g powder sample was taken on a sieve of 0.05 mm, and the sample was vibrated on the sieve for 10 minutes. The percentage of particles below 50 microns (% Pf) was determined.
5. *HOMOGENEITY INDEX* (Ph Eur 2.9.12) [1]: A 100 g powder sample was assessed by subjecting a sieve stack to vibration for 10 minutes at a speed of 10 using a sieve vibrator. The sieves of 0.355, 0.212, 0.100, and 0.05 mm were utilized for the test. The amount of product that remained on each sieve and the quantity that passed through the 0.05 mm sieve was determined. The previously determined percentage of fine particles (<50 μ) was considered. Subsequently, the acquired data was subjected to the following equation to calculate the homogeneity index (Iθ):


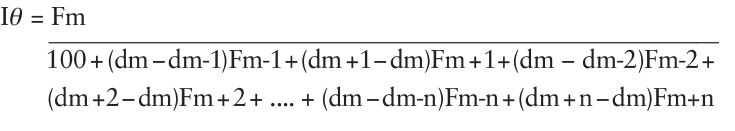


where Iθ = relative homogeneity index; Fm = percentage of particles in the majority range; Fm − 1 = percentage of particles in the range immediately below the majority range; Fm + 1 = percentage of particles in the range immediately above the majority range; n = order number of the fraction under study, within a series, with respect to the majority fraction; dm = the mean diameter of particles in the majority fraction; dm − 1 = the mean diameter of particles in the fraction of the range immediately below the majority range; dm + 1 = mean diameter of the particles in the fraction of the range immediately above the majority range

1. *EFFERVESCENCE* [1, 3]: A tablet was placed in a vessel containing 200 ml of purified water at 15 - 25°C. Time was recorded for the tablet of the sample powder to get dispersed, and no agglomerates remained. The specified time is within 5 minutes.
2. *DISINTEGRATION WITH DISC* [1, 3]: Inside the mouth, mechanical disintegration takes place, which is mimicked by an in-vitro test of disintegration with disc and disintegration without disc. The time specified in pharmacopeia is within 3 minutes (≤ 3 minutes).
3. *DISINTEGRATION WITHOUT DISC* [1, 3]: It is the same as the “disintegration with disc” test. The specification is similar to the disintegration test with the disc, i.e., ≤ 3 minutes.

REFERENCES

1. Council of Europe. European Pharmacopoeia (Ph. Eur.). 10th ed. Strasbourg Cedex, France: European Directorate for the Quality of Medicines & HealthCare of the Council of Europe (EDQM); 2019.

2. Singh I, Thakur AK, Bala R, Madan R. SeDeM expert system, an innovative tool for developing directly compressible tablets: a review. Current Drug Research Reviews Formerly: Current Drug Abuse Reviews. 2021;13(1):16-24.

3. United States Pharmacopeial Convention. USP 44 NF 39: U. S. Pharmacopoeia National Formulary: United States Pharmacopeial Convention; 2021.
